# Galectin-receptor interaction: a key player in liver fibrosis induced by *Schistosoma japonicum* infection

**DOI:** 10.1186/s13071-024-06314-5

**Published:** 2024-05-20

**Authors:** Ziyun Huang, Xingzhuo Liu, Shiguang Huang, Fangli Lu

**Affiliations:** 1 Department of Parasitology, Zhongshan School of Medicine, Sun Yat-sen University, Guangzhou, China; 2 Shenzhen Nanyou Malt Dentistry Out-Patient Department, Shengzhen, China; 3 School of Stomatology, Jinan University, Guangzhou, China; 4 Scientific Research Center, The Seventh Affiliated Hospital of Sun Yat-sen University, Shenzhen, China; 5 Department of Parasitology, School of Medicine, Sun Yat-sen University, Shenzhen, China; 6 Key Laboratory of Tropical Disease of the Ministry of Education, Sun Yat-sen University, Guangzhou, China

**Keywords:** *Schistosoma japonicum*, Galectin-receptor interactions, Liver fibrosis, Macrophage autophagy, Mice

## Abstract

**Background:**

*Schistosoma japonicum* eggs lodge in the liver and induce a fibrotic granulomatous immune response in the liver of host. Galectin 3 (Gal-3) is a protein implicated in fibrosis in multiple organs. However, the pathology and molecular mechanisms promoting hepatic granuloma formation remain poorly understood.

**Methods:**

To investigate the effect of blocking galectin-receptor interactions by α-lactose on liver immunopathology in mice with *S. japonicum* infection, C57BL/6 mice were infected with *S. japonicum* and alpha (α)-lactose was intraperitoneally injected to block the interactions of galectins and their receptors.

**Results:**

Compared with *S. japonicum*-infected mice, there were significantly decreased Gal-3 mRNA and protein expression levels, decreased intensity of Gal-3 fluorescence in the liver, decreased serum ALT and AST levels, decreased egg numbers of *S. japonicum* in the liver section, attenuated hepatic and spleen pathology, and alleviated liver fibrosis accompanied with decreased protein expression levels of fibrosis markers [α-smooth muscle actin (α-SMA), collagen I, and collagen IV] in the liver of *S. japonicum*-infected mice blocked galectin-receptor interactions with hematoxylin-eosin staining, Masson’s trichrome staining, immunohistochemistry, or Western blot analysis. Compared with *S. japonicum*-infected mice, blocking galectin-receptor interactions led to increased eosinophil infiltration and higher eosinophil cationic protein (ECP) expression in the liver, accompanied by increased mRNA levels of eosinophil granule proteins [ECP and eosinophil peroxidase (EPO)], IL-5, CCL11, and CCR3 in the liver and decreased mRNA levels of Gal-3 and M2 macrophage cytokines (TGF-β, IL-10, and IL-4) in the liver and spleen by using quantitative real-time reverse transcription-polymerase chain reaction. In addition, there were increased Beclin1 protein expression and protein expression ratio of LC3B-II/LC3B-I and decreased p62 protein expression and protein expression ratios of phospho-mTOR/mTOR and phospho-AKT/AKT by Western blot; increased double-labeled F4/80^+^/LC3B^+^ cells by immunofluorescence staining; increased M1 macrophage polarization in the liver of *S. japonicum*-infected mice blocked galectin-receptor interactions by flow cytometric analysis and immunofluorescence staining.

**Conclusions:**

Our data found that blockage of galectin-receptor interactions downregulated Gal-3, which in turn led to reduced liver functional damage, elevated liver eosinophil recruitment, promoted macrophage autophagy through the Akt/mTOR signaling pathway, and alleviated liver pathology and fibrosis. Therefore, Gal-3 plays a pivotal role during *S. japonicum* infection and could be a target of pharmacologic potential for liver fibrosis induced by *S. japonicum* infection.

**Graphical Abstract:**

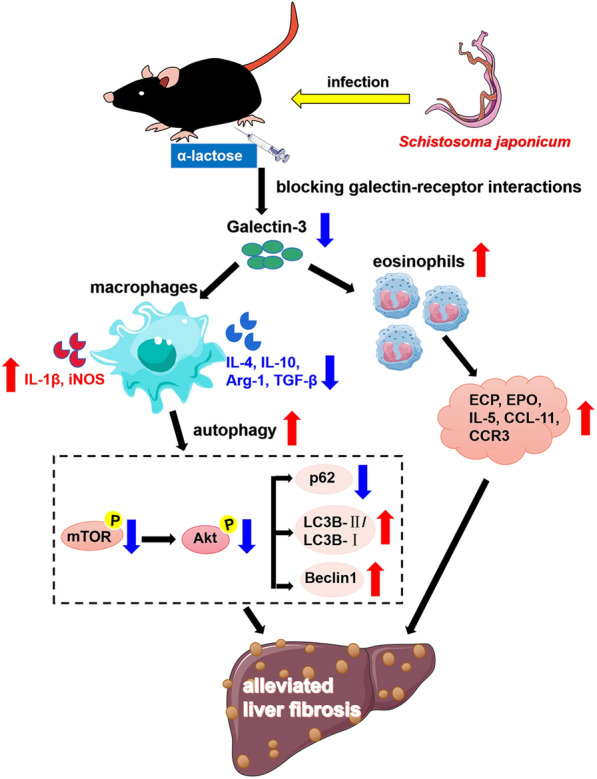

**Supplementary Information:**

The online version contains supplementary material available at 10.1186/s13071-024-06314-5.

## Background

Schistosomiasis is one of the serious tropical diseases caused by three main species of schistosomes: *Schistosoma mansoni*, *S. haematobium*, and *S. japonicum* [1]. According to the World Health Organization report, at least 251.4 million people required preventive treatment for schistosomiasis in 2021, out of which > 75.3 million people were reported to have been treated [2]. *Schistosoma japonicum* infection causes granulomatous responses to parasite eggs trapped in the liver, resulting in severe liver fibrosis and irreversible impairment of the liver and even death of the host [3]. So far, the mechanism of egg-induced liver fibrosis in *S. japonicum* infection remains unclear. Therefore, exploring the aspects of immunology and interplay between host and parasites during *S. japonicum* infection may have essential roles in schistosomiasis japonica-induced liver fibrosis.

Galectins are a family of carbohydrate-binding proteins that are expressed by a wide variety of cells and bind to galactose-containing glycans; they are involved in many physiological functions such as inflammation, immune responses, cell migration, autophagy, and signaling, and 15 members have been identified in various cells and tissues [4]. Galectin (Gal)-1 and Gal-3 facilitate the proliferation of hepatic stellate cells and play an important role in liver fibrosis [5], in which Gal-1 is a promising molecular target for the development of therapeutic tools to treat fibrosis in different chronic diseases [6]. Gal-3 is a chimeric lectin and potent driver of many aspects of fibrosis, and Gal-3 binds to *Schistosoma* egg antigens and promotes liver fibrosis of *S. mansoni*-infected C57BL/6 mice [7]. Our previous study found that Gal-1, Gal-3, eosinophils, and macrophages may participate in the development of egg granulomatous inflammation and fibrosis induced by *S. japonicum* infection [8]; however, the mechanisms remain unknown.

Macrophage autophagy has a critical regulatory function in downregulation of liver immunopathology in different kinds of diseases. It has been reported that *S. japonicum* egg antigen-triggered macrophage autophagy limited the development of pathology in the liver of C57BL/6 mice [9]. Galectins expressed by immune cells can participate in host responses to infection by directly binding to microorganisms or by modulating antimicrobial functions such as autophagy [10]. However, the role of galectins in regulating liver fibrosis caused by *S. japonicum* infection remains poorly understood, and little is known about whether and how galectins can impact macrophage autophagy and the liver immunopathology after schistosome infection. Elucidating the molecular mechanisms that promote or restrict tissue fibrosis may lead to more effective strategies for immunological intervention in schistosomiasis. By blocking galectin-receptor interactions with α-lactose, our data demonstrated that Gal-3, eosinophils, macrophages, and autophagy may play important roles in liver pathology and fibrosis caused by *S. japonicum* infection. Therefore, Gal-3 could be a target for early therapy of hepatic fibrosis induced by *S. japonicum* infection.

## Methods

### Ethics statement

All the experimental animals were housed in groups of five and given 5 days to acclimate to the housing facility. The mice were housed in an air-conditioned room (22–25 °C), maintained under a 12-h light-dark cycle, and water and food were supplied ad libitum. The health of mice was monitored twice daily, and no adverse events were observed during feeding. All surgeries were performed under anesthesia using isoflurane, and the appropriate organs were harvested when mice were killed.

### *Schistosoma japonicum* and experimental infection

Female, 6–8-week-old C57BL/6 mice were obtained from the Animal Center of Sun Yat-sen University (Guangzhou, China). *Oncomelania hupensis* snails were purchased from the National Institute of Parasitic Diseases, Chinese Center for Disease Control and Prevention (Shanghai, China). A total of 40 C57BL/6 mice were used in this study, which were randomly assigned to 4 groups of 10 in each group: (i) naive group: mice were intraperitoneal (i.p.) injected with PBS daily as uninfected control; (ii) α-lactose-treated group (lact group): mice were i.p. injected with α-lactose (L8040-100, Sigma-Aldrich, St. Louis, MO, USA) solution in PBS alone as α-lactose control; (iii) *S. japonicum*-infected group (*Sj* group): mice were percutaneously infected with 30 cercariae of *S. japonicum*, and (iv) *S. japonicum* + α-lactose group (*Sj* + lact group): mice were percutaneously infected with 30 cercariae of *S. japonicum* and i.p. injected with α-lactose treatment. Because male-female adult parasites pairing is common at 18 days after *S. japonicum* infection in mouse models [11], lact group and *Sj* + lact group were administered 1.5 mM of α-lactose PBS solution daily from 19 days post infection (p.i.) to 8 weeks p.i., while naive group was i.p. injected with equal volume of PBS daily. At week 8 post-*S. japonicum* infection, each group of mice was i.p. anesthetized with isoflurane and weighed, blood samples were drawn from the orbit to determine serum levels of aspartic aminotransferase (AST) and alanine aminotransferase (ALT), and livers and spleens were harvested, weighed, and then stored based on different experimental needs.

### Measurement of serum enzyme activities

The whole blood samples of mice were collected and kept at room temperature for 2 h and then centrifuged at 3000 rpm for 10 min after coagulation. The supernatant was harvested, and the serum levels of AST and ALT were measured to evaluate liver function on an automatic biochemical analyzer (Chemray 800; Rayto Life and Analytical Sciences Co., Shenzhen, China) by different assay kits (Rayto Life and Analytical Sciences Co.) according to the manufacturer’s instructions.

### Determination of liver and spleen indexes

The body weight of each mouse in different groups was measured before they were killed, and the livers and spleens were excised and weighed. Liver index = liver weight (g)/body weight (g) × 100%, and spleen index = spleen weight (g)/body weight (g) × 100%.

### Histopathological examination

The liver and spleen of mice were collected, and part of the tissues was formaldehyde-fixed in 10% buffered natural formaldehyde (Guangzhou Chemical Reagent Factory, Guangzhou, China) for over 48 h, paraffin-embedded, and sectioned. Tissue sections (4 µm) were stained with hematoxylin-eosin (H&E) or Masson’s trichrome solution (Wuhan Servicebio Technology Co., Ltd., Wuhan, China). A semi-quantitative scoring system was used to evaluate histological changes in liver tissues from four mice of each group. The histopathological changes were determined under 200 × magnification in five noncontiguous sections. Microscopic scores of the inflammation severity were graded into four grades: 0, no inflammation; 1, mild inflammation; 2, moderate inflammation and necrosis, 3, severe inflammation and necrosis [12]. The eosinophil numbers in liver tissues were quantified using images taken with a digital camera system under 1000 × magnification measured using Image Pro plus 6.0 software (Media Cybernetics, Inc., MD, USA). The egg numbers of *S. japonicum* in liver sections were counted in images taken with a digital camera system under 200 × magnification. Eosinophil density was expressed as the number of eosinophils per square millimeter in the liver. Collagen appears blue using Massonʼs trichrome staining, and the blue area reflects the amount of collagen. The percentage of the area stained with blue color in total area was estimated quantitatively by using Image Pro Plus 6.0 software (Media Cybernetics, Inc.) under 100 × magnification [13], and data are expressed in units of area. The severity of fibrosis was distinguished by the area of collagen.

### Immunohistochemistry

The frozen liver tissue was cut into 10-µm sections (50 mm distance between sections) using a freezing microtome. Liver sections were pretreated with 0.3% H_2_O_2_ at room temperature for 30 min. After blocking with 5% bovine serum albumin (BSA) in PBS (pH = 7.4) for 30 min, the sections were incubated overnight at 4 °C with primary antibody for routine immunohistochemistry. The primary antibodies used were rabbit anti-Gal-9 (1:50, Proteintech, Wuhan, China), rabbit anti-Gal-3 (1:50, Proteintech), rabbit anti-Gal-1 (1:50, Proteintech), rabbit anti-collagen IV (1:500, Abcam Plc., Cambridge, UK), rabbit anti-collagen I (1:200, Affinity Biosciences Ltd., Jiangsu, China), and rabbit anti-alpha-smooth muscle actin (α-SMA, 1:200, Affinity Biosciences Ltd.). After rinsing, the sections were incubated with a streptavidin-biotin-peroxidase complex kit and then immersed in enhanced 3,3′—diaminobenzidine (Wuhan Servicebio Technology Co., Ltd.) to visualize the staining. Finally, the sections were counterstained with hematoxylin and then dehydrated, cleared, and cover-slipped. The liver sections from each mouse of different groups were analyzed. The immunoreactive areas were quantified using images taken with a digital camera system at 400 × magnification and analyzed with an image analysis program (ImageJ v1.8.0, the National Institutes of Health, Bethesda, MD, USA).

To perform double-labeled immunofluorescence staining, liver sections were incubated overnight at 4 °C with rat anti-F4/80 (1:200, Santa Cruz Biotechnologys, Shanghai, China) and rabbit anti-MHC Class II (MHC-II, 1:200, Beijing Bioss Biotechnology Co., Ltd., Beijing, China) or with rat anti-F4/80 (1:200, Santa Cruz Biotechnologys) and rabbit anti-CD206 (1:200, Affinity Biosciences Ltd.). The sections were then incubated with anti-rat fluorescent IgG (1:400, AF488, Cell Signaling Technology Inc., Massachusetts, USA) and anti-rabbit fluorescent IgG (1:400, AF594, Cell Signaling Technology Inc.) at room temperature for 1 h and stained with 4,6-diamidino-2-phenylindole (DAPI). In addition, the liver sections were incubated overnight at 4 °C with rabbit anti-LC3B (1:200, Proteintech) and rat anti-F4/80 (1:200, Santa Cruz Biotechnologys), with rabbit anti-LC3B (1:200, Proteintech) and rabbit anti-Gal-3 (1:100, Proteintech), and with rabbit anti-Gal-3 (1:100, Proteintech) and rabbit anti-ECP (eosinophil cationic protein, 1:100, Proteintech), respectively. The sections were then incubated with anti-rabbit fluorescent IgG (1:400, AF488, Cell Signaling Technology Inc., Massachusetts, USA), anti-rat fluorescent IgG (1:400, AF594, Thermo Fisher Scientific Inc., Massachusetts, USA), and anti-rabbit fluorescent IgG (1:400, AF594, Cell Signaling Technology Inc.) at room temperature for 1 h, respectively, and then stained with 4,6-diamidino-2-phenylindole (DAPI). Immunofluorescence images were captured by confocal microscope Nikon C2 (Nikon Corp., Tokyo, Japan). The numbers of F4/80 and MHC-II, F4/80 and CD206, and F4/80 and LC3B double-stained immunoreactive cells were counted in five randomly selected areas (0.01 mm^2^ each) in each liver section. The analysis of morphological studies was performed by two blinded researchers.

### Flow cytometry

Liver tissue from each mouse was cut into small pieces, and liver leukocytes were separated by Ficoll-Hypaque (Haoyang Biological Manufacture Co., Ltd., Tianjin, China) gradient centrifugation after being filtered through a 70-µm cell sieve. Spleen tissue from each mouse was also rendered into single-cell suspensions by dispersing through a 70-µm cell sieve. The cell suspension was purified with ACK lysis buffer, and cell concentration was adjusted appropriately. For flow cytometry analysis, liver leukocytes and spleen lymphocytes were washed twice and suspended in pre-chilled PBS with 0.1% BSA. A total of 1 × 10^6^ cells/100 µl were incubated with PE/Cyanine7-conjugated anti-CD45 antibody, EV450-conjugated anti-F4/80 antibody, APC-conjugated anti-CD11b antibody, PE-conjugated anti-MHC-II antibody, and Ghost Dye™ Red 780 (ELGBIO Biotechnology Co., Ltd., Guangzhou, China) for surface staining, and then the cells were re-suspended with fixation/permeabilization buffer (BD Biosciences Inc., New York, USA) and incubated with FITC-conjugated anti-CD206 antibody (ELGBIO Biotechnology Co., Ltd.) for intracellular staining. Data were detected with a CytoFLEX flow cytometer and analyzed using FlowJo software (Treestar Inc., CA, USA).

### Determination of mRNA expression by using quantitative real-time reverse transcription-polymerase chain reaction (qRT-PCR)

Total RNA was isolated from liver and spleen tissues using FastPure® Cell/Tissue Total RNA Isolation Kit (Vazyme Biotech Co., Nanjing, China) according to the manufacturer’s instructions. Then, cDNA was synthesized from total RNA using PrimeScript^™^ II 1st Strand cDNA Synthesis Kit (TaKaRa Bio Inc., Shiga, Japan). To determine the mRNA levels of Gal-1, Gal-3, Gal-9, eosinophil cationic granule proteins [ECP, eosinophil peroxidase (EPO)], and eosinophil chemokines [IL-5, C–C motif chemokine 11 (CCL11), and CC chemokine receptor3 (CCR3)] in the liver tissue, and the mRNA levels of M1 macrophage-associated genes (iNOS and IL-1β) and M2 macrophage-associated genes (TGF-β, Arg1, IL-4, and IL-10) in the liver and spleen tissues, qRT-PCR was performed in a 10 µl volume with the SYBR Green qPCR Master Mix (TaKaRa Bio Inc.). The PCR was run on a CFX96 Touch^®^ Real-Time PCR Detection System (Bio-Rad Laboratories, CA, USA) for 30 s at 95 °C, followed by 39 cycles of 5 s at 95 °C and 30 s at 60 °C. Then, the melting curve was analyzed (95 °C for 15 s and 65 °C for 15 s). Relative gene expression levels were determined using the 2^−ΔΔCt^ method with Light Cycler 480 software (Roche, version 1.5.0). Mouse housekeeping gene, GAPDH, was used as the control to normalize specific mRNA expression levels. The fold change in the expression of each target gene mRNA relative to GAPDH in each group was determined using the 2.^−ΔΔCt^ method. All primers are listed in Table 1.

**Table 1:** Primer sequences used for quantitative real-time reverse transcription-polymerase chain reaction

Gene	Forward primer (5′—3′)	Reverse primer (5′—3′)	Accession
GAPDH	ACTCCACTCACGGCAAATTC	TCTCCATGGTGGTGAAGACA	NM_001289726.1
Gal-1	CGCCAGCAACCTGAATC	GTCCCATCTTCCTTGGTGTTA	NM_008495.2
Gal-3	GCTACTGGCCCCTTTGGT	CCAGGCAAGGGCATATCGTA	NM_001145953.1
Gal-9	GCAGGAGGGACTTCAGGTGA	GCCCCCACTGTCCGTTCT	NM_001159301.1
EPO	CTGTCTCCTGACTAACCGCTCT	TCAGCGGCTAGGCGATTGTGTT	XM_006532174.3
ECP	CATCACCAGTCGGAGGAGAACA	ATGGGACTGTCCTGTGGAGTTC	XM_021155370.1
IL-5	GATGAGGCTTCCTGTCCCTACT	TGACAGGTTTTGGAATAGCATTTCC	NM_010558.1
CCL11	TCCATCCCAACTTCCTGCTGCT	CTCTTTGCCCAACCTGGTCTTG	NM_011330.3
CCR3	CCACTGTACTCCCTGGTGTTCA	GGACAGTGAAGAGAAAGAGCAGG	XM_017313120.2
iNOS	GTTCTCAGCCCAACAATACAAGA	GTGGACGGGTCGATGTCAC	NM_010927.4
IL-1β	AATGACCTGTTCTTTGAAGTTGA	TGATGTGCTGCTGCGAGATTTGAAG	NM_008361.4
TGF-β	TGATACGCCTGAGTGGCTGTCT	CACAAGAGCAGTGAGCGCTGAA	XM_021167684.1
Arg1	CAGAAGAATGGAAGAGTCAG	CAGATATGCAGGGAGTCACC	NM_007482.3
IL-4	ACAGGAGAAGGGACGCCAT	GAAGCCCTACAGACGAGCTCA	NM_021283.2
IL-10	AGCCGGGAAGACAATAACTG	CATTTCCGATAAGGCTTGG	NM_010548.2

*ARG-1* Arginase 1, *CCL11 C* C motif chemokine 11, *CCR3 CC* chemokine receptor 3, *ECP* eosinophil cationic protein, *EPO* eosinophil peroxidase, *GAL* galectin, *GAPDH* glyceraldehyde 3-phosphate dehydrogenase, *iNOS* inducible nitric oxide synthase, *IL* interleukin, *TGF-β* transforming growth factor beta

### Western blot assay

Hepatic tissues were extracted and lysed in RIPA lysis buffer (Shanghai Beyotime Biotechnology Co., Ltd., Shanghai, China) and then ultrasonicated and centrifuged at 10, 000 × g for 5 min at 4 °C. The supernatants were collected, and the protein concentrations were quantified via BCA Protein Assay Kit (Shanghai Beyotime Biotechnology Co., Ltd.) and then boiled in SDS-PAGE loading buffer at 100 °C for 10 min. An equal amount of 20 μg protein from each sample was electrophoresed on 10% SDS-page gels (Shanghai Epizyme Biomedical Technology Co., Ltd., Shanghai, China) and transferred onto a PVDF membrane (Millipore, Massachusetts, USA). Next, the membranes were blocked with Protein Free Rapid Blocking Buffer (Shanghai Epizyme Biomedical Technology Co., Ltd.) for 15 min and then incubated with shaking overnight at 4 °C with the following primary antibodies: rabbit anti-Gal-9 (1:50, Proteintech, Wuhan, China), rabbit anti-Gal-3 (1:50, Proteintech), rabbit anti-Gal-1 (1:50, Proteintech), rabbit anti-collagen IV (1:1000, Abcam Plc.), rabbit anti-collagen I (1:1000, Affinity Biosciences Ltd.), rabbit anti-α-SMA (1:1000, Affinity Biosciences Ltd.), rabbit anti-Phospho-mTOR (1:1000, Affinity Biosciences Ltd.), rabbit anti-mTOR (1:1000, Affinity Biosciences Ltd.), rabbit anti-Phospho-AKT (1:1000, Affinity Biosciences Ltd.), rabbit anti-AKT (1:1000, Affinity Biosciences Ltd.), rabbit anti-SQSTM1/p62 (1:1000, Abcam Plc.), rabbit anti-Beclin1 (1:1000, Proteintech Group Inc., Rosemont, USA), rabbit anti-LC3B (1:1000, Abcam Plc.), and rabbit anti-GAPDH (1:1500, Cell Signaling Technology Inc.), respectively. The membranes were then incubated with goat-anti-rabbit IgG antibodies (1:10,000, Cell Signaling Technology Inc.) for 1 h at room temperature. Protein bands were visualized using the chemiluminescence imaging system (Bio-Rad Laboratories Inc.) and quantified using ImageJ v1.8.0 (the National Institutes of Health).

### Statistical analysis

All data are presented as the mean ± standard deviation (SD) and were analyzed using SPSS 20.0 software to test normality. SPSS 20.0 software was used to conduct *t*-test of independent samples (for comparison between two groups of data) or one-way ANOVA (for comparison between multiple groups of data) for statistical analysis. All histograms were designed with GraphPad Prism 8 (GraphPad software, San Diego, CA, USA). *P* < 0.05 was considered statistically significant.

## Results

### Blockage of galectin-receptor interactions decreased Gal-3 level in the liver of *S. japonicum*-infected mice

**Fig. 1:**
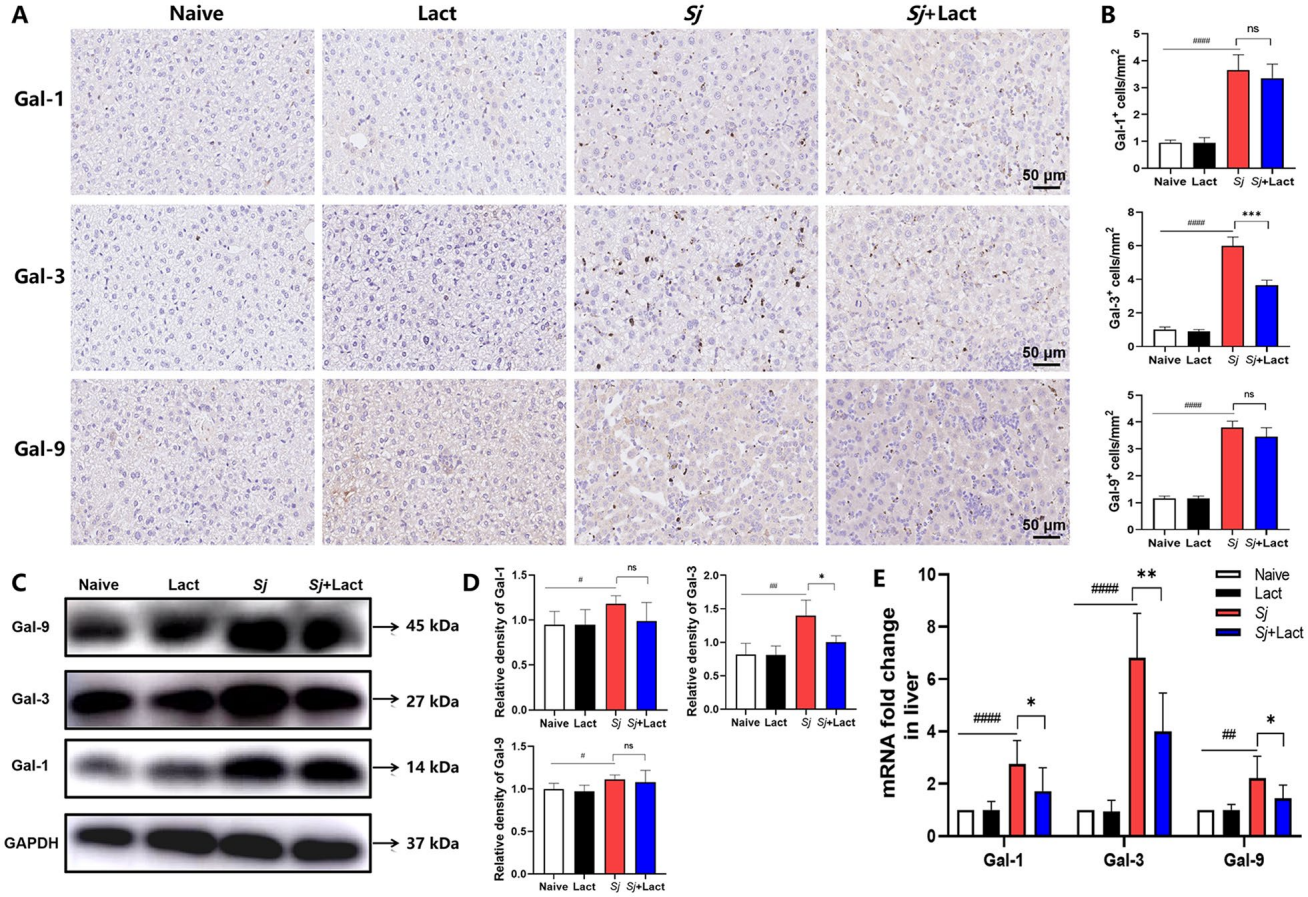
The expression of Gal-1, Gal-3, and Gal-9 in the livers of different groups of mice. **A** Immunohistochemical staining of Gal-1, Gal-3, and Gal-9 positive cells in the livers of different groups of mice. Original magnification 400 × (scale bar = 50 µm). **B** The numbers of Gal-1, Gal-3, and Gal-9 positive cells were counted in five random fields to quantify them. Data are expressed as mean ± SD (*n* = 4). **C** The protein expression levels of Gal-1, Gal-3, and Gal-9 in the liver were detected by Western blot. **D** Densitometric analysis of Gal-1, Gal-3, and Gal-9 normalized to the endogenous control (GAPDH) and expressed as fold change. Data are expressed as mean ± SD (*n* = 4). **E** The mRNA expression levels of Gal-1, Gal-3, and Gal-9 in the liver was detected by qRT-PCR (*n* = 8). ^##^*P* < 0.01, ^###^*P* < 0.001, ^####^*P* < 0.0001, *Sj* group compared with naive group and lact group; ^*^P < 0.05, ^**^P < 0.01, and ^***^P < 0.001, *Sj* + lact group compared with *Sj* group

Only a few Gal-1, Gal-3, and Gal-9 positive cells labeled dark brown were observed in the liver section of naive group and lact group. However, there were many Gal-1, Gal-3, and Gal-9 positive cells in the liver section of *Sj* group (Fig. 1A). Compared with uninfected controls, there were significantly increased Gal-1, Gal-3, and Gal-9 positive cells in the liver of *Sj* group (*P* < 0.0001). Compared with *Sj* group, there were significantly decreased Gal-3 positive cells in the liver of *Sj* + lact group (*P* < 0.001); however, the numbers of Gal-1 or Gal-9 positive cells had no statistical significance (*P* > 0.05, Fig. 1B) (ANOVA; *F*_Gal-9_ = 173.667, *F*_Gal-3_ = 239.084, *F*_Gal-1_ = 53.449; *P* < 0.0001). Consistent results were also found by Western blot; only Gal-3 protein expression was significantly decreased in the liver of *Sj* + lact group compared to *Sj* group (*P* < 0.05, Fig. 1C, 1D).

qRT-PCR assay showed that compared with naive group and lact group, the mRNA levels of Gal-1, Gal-3, and Gal-9 were significantly increased in the liver of both *Sj* group (*P* < 0.0001, *P* < 0.0001, and *P* < 0.01, respectively) and *Sj* + lact group (*P* < 0.05, *P* < 0.0001, and *P* < 0.05, respectively) (ANOVA; *F*_Gal-1_ = 12.677, *F*_Gal-3_ = 48.817, *F*_Gal-9_ = 10.284; *P* < 0.0001). Compared with *Sj* group, Gal-3 level was significantly decreased in the liver of *Sj* + lact group (*P* < 0.01, Fig. 1E). These findings suggest that Gal-3 is strongly involved in hepatic immunopathology induced by *S. japonicum* infection.

### Blockage of galectin-receptor interactions relieved liver function damage of *S. japonicum*-infected mice

**Fig. 2:**
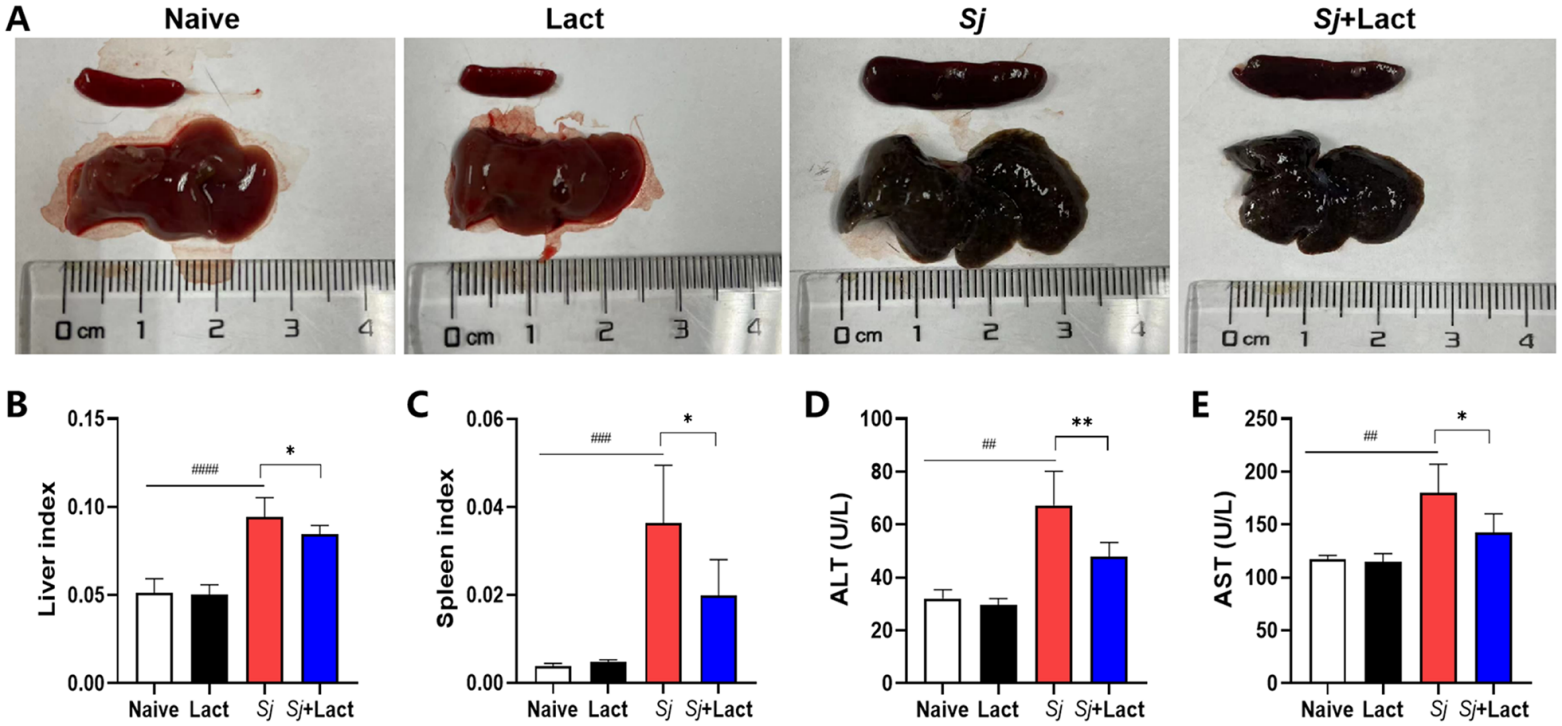
Blockage of galectin-receptor interactions alleviated the liver function damage of *S. japonicum*-infected mice. **A** Gross specimens of liver and spleen of different groups of mice. **B** The liver indexes of different groups of mice. **C** The spleen indexes of different groups of mice. **D** Serum ALT levels of different groups of mice. **E** Serum AST levels of different groups of mice. Results are expressed as mean ± SD (*n* = 7). ^##^*P* < 0.01, ^###^*P* < 0.001, ^####^*P* < 0.0001, *Sj* group compared with naive group and lact group; ^*^P < 0.05, ^**^P < 0.01, *Sj* + lact group compared with *Sj* group

Gross observation of liver specimens found that the livers of normal control mice (either naive mouse or α-lactose-treated mouse) were light red color with a smooth surface; in contrast, the livers of *S. japonicum*-infected mice (either *Sj* mouse or *Sj* + lact mouse) were dark red color with numerous irregular whitish spots on the surface, and the spleens were markedly enlarged and darkened (Fig. 2A).

The liver and spleen indexes of *Sj* group and *Sj* + lact group were significantly higher than those of naive group (*P* < 0.0001 and *P* < 0.001, respectively) and lact group (*P* < 0.0001 and *P* < 0.001, respectively). Compared with *Sj* group, the liver and spleen indexes of *Sj* + lact group were significantly increased (*P* < 0.05) (ANOVA; *F*_liver indexes_ = 92.410, *F*_spleen indexes_ = 50.484; *P* < 0.0001, Fig. 2B, C). The results suggested that blockage of galectin-receptor interaction was beneficial to alleviate liver and spleen damage induced by chronic *S. japonicum* infection.

Serum levels of ALT and AST are used to assess liver function. Compared with naive group and lact group, serum levels of ALT and AST were significantly increased in *Sj* group (*P* < 0.01) and *Sj* + lact group (*P* < 0.05) (ANOVA; *F*_ALT_ = 41.679, *F*_AST_ = 23.687; *P* < 0.0001). Compared with *Sj* group, serum ALT and AST levels were significantly decreased in *Sj* + lact group (*P* < 0.01 and *P* < 0.05, respectively, Fig. 2D, E).

### Blockage of galectin-receptor interactions promoted eosinophil infiltration in the liver and attenuated the pathology of liver and spleen of *S. japonicum*-infected mice

**Fig. 3:**
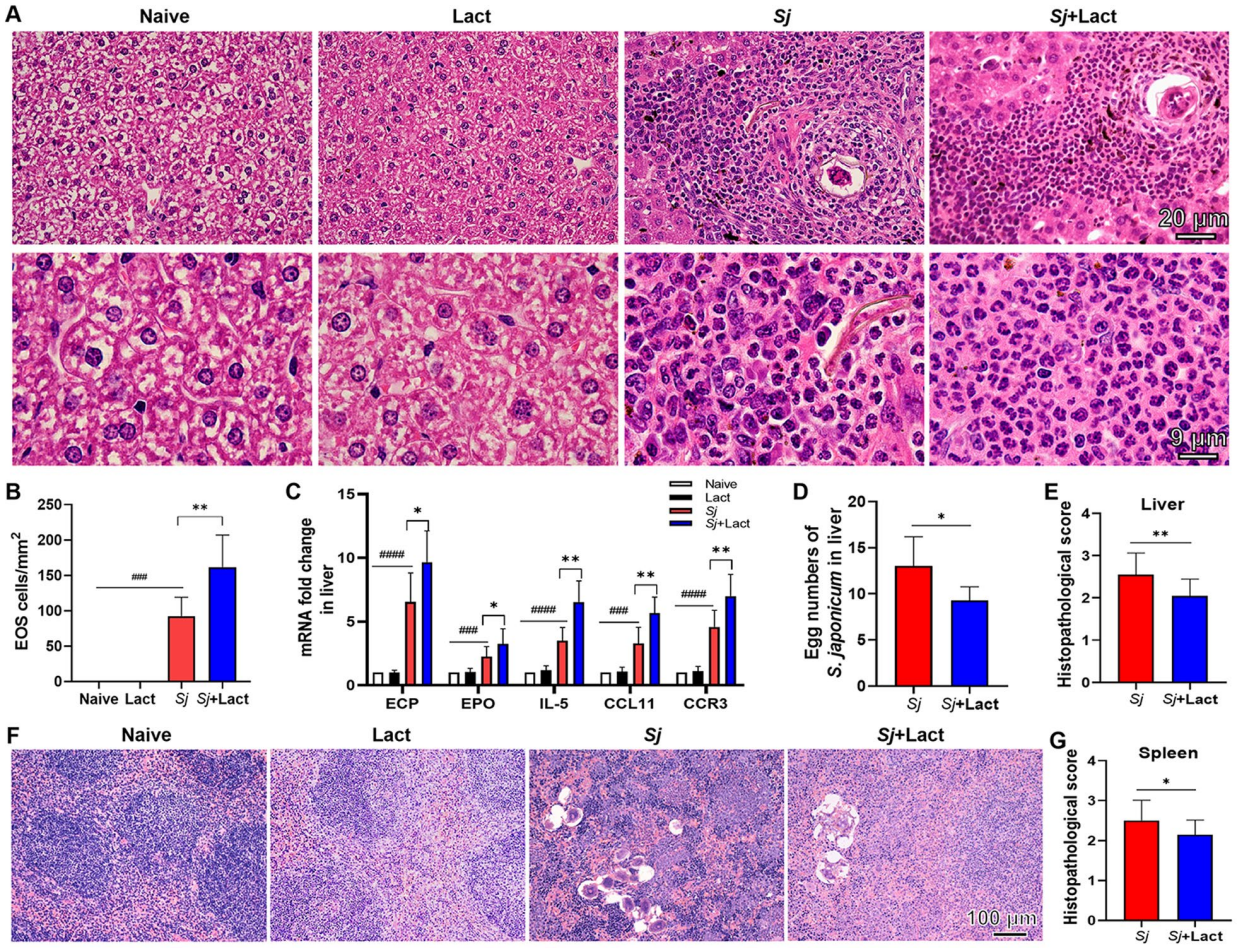
The liver and spleen histopathology of different groups of mice. Mice were infected with 30 *Schistosoma japonicum* cercariae and killed at 8 weeks p.i. **A** Representative microscopic pictures of the liver sections of different groups stained with H&E. No histological change was observed in the liver and spleen of naive group and lact group; egg granulomas and a large number of eosinophils around *S. japonicum* egg granulomas were observed in the liver and spleen of *Sj* group and *Sj* + lact group. Original magnifications of the images are 400 × (scale bar = 20 µm) and 1000 × (scale bar = 9 µm). **B** Eosinophil count analysis in the liver tissues. Data are represented as mean ± SD. There were six mice per group. **C** The mRNA expressions of ECP, EPO, IL-5, CCL11, and CCR3 in the livers of different groups of mice at 8 weeks p.i. Values are means from triplicate measurements, and data are presented as means ± SD (*n* = 10). **D** The egg numbers of *S. japonicum* in the livers. Data are represented as mean ± SD (*n* = 5). **E** Histopathological score analysis of the liver tissues. **F** Representative microscopic pictures of the spleen sections of different groups of mice stained with H&E. **G** Histopathological score analysis of the spleen tissues. Original magnification 200 × (scale bar = 100 µm). Data are represented as mean ± SD (*n* = 4). ^###^*P* < 0.001 and ^####^*P* < 0.0001, *Sj* group compared with naive group and lact group; ^*^P < 0.05 and ^**^P < 0.01, *Sj* + lact group compared with *Sj* group

The livers and spleens of different groups of mice were examined histologically at 8 weeks p.i. Compared with uninfected controls, severe inflammation and significantly increased eosinophil infiltration around egg granulomas were observed in the liver sections of *Sj* group and *Sj* + lact group (Fig. 3A). The numbers of eosinophils in liver sections of different groups were counted under 1000 × magnification. Quantitative analysis showed that compared with naive group or lact group, the numbers of eosinophils were significantly increased in the livers of *Sj* group (*P* < 0.001) and *Sj* + lact group (*P* < 0.001), and eosinophil number was significantly increased in the liver of *Sj* + lact group compared with *Sj* group (*P* < 0.01, Fig. 3B) (ANOVA; *F*_ALT_ = 41.679, *F*_AST_ = 23.687; *P* < 0.0001).

The mRNA expression levels of eosinophil granule proteins (ECP and EPO) and eosinophil chemokines (IL-5, CCL11, and CCR3) in the liver were determined. Compared with naive group or lact group, the mRNA levels of ECP (*P* < 0.0001), EPO (*P* < 0.001 and *P* < 0.0001, respectively), IL-5 (*P* < 0.0001), CCL11 (*P* < 0.001 and *P* < 0.0001, respectively), and CCR3 (*P* < 0.0001) were significantly increased in the livers of *Sj* group and *Sj* + lact group. Compared with *Sj* group, the mRNA levels of ECP (*P<0.05*), EPO (*P<0.05)*, IL-5 (*P<0.01*), CCL11 (*P<0.01)*, and CCR3 (*P<0.01)* were significantly increased in the liver of *Sj* + lact group ( Fig. 3C) (ANOVA; *F*_IL-5_ = 40.570, *F*_ECP_ = 60.928, *F*_EPO_ = 14.805, *F*_CCL11_ = 47.381, *F*_CCR3_ = 42.927; *P* < 0.0001). The results indicated that blockage of galectin-receptor interactions increased eosinophil infiltration in the liver of *S. japonicum*-infected mice.

Histological observation showed that no pathological change was observed in the liver and spleen tissues of naive group and lact group. However, severe histological changes were observed in the liver and spleen tissues, and diffuse *S. japonicum* egg granulomas were surrounded by many inflammatory cells in the liver and spleen tissues of *Sj* group, while attenuated granulomatous inflammation surrounding egg granulomas was observed in the liver (Fig. 3A) and spleen (Fig. 3F) tissues of *Sj* + lact group. Furthermore, the egg numbers of *S. japonicum* in the liver sections of *Sj* + lact group was lower than in *Sj* group (*t*-test, *t*_liver_ = 2.367, *P* < 0.05, Fig. 3D). Compared with *Sj* group, semi-quantitative histopathological scores based on histopathological changes in the liver (Fig. 3E) and spleen (Fig. 3G) tissues were significantly decreased in *Sj* + lact group (*t*-test, *t*_liver_ = 3.468, *P* < 0.01 and *t*_spleen_ = 2.483, *P* < 0.05, respectively). Therefore, our data demonstrated a profound protective role of eosinophils against *S. japonicum*; however, the molecular mechanism had not been elucidated.

**Fig. 4:**
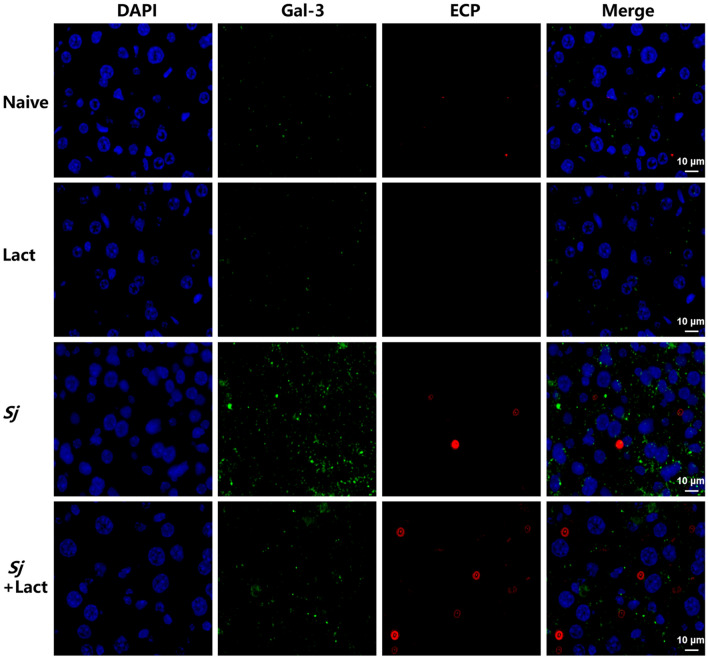
Double immunofluorescence staining of Gal-3 and ECP in the liver tissues of different groups of mice. Fluorescence microscopy observation of liver tissues in different groups of mice stained with anti-Gal-3 (green) and anti-ECP (eosinophils marker, red). Tissues were counterstained with DAPI (blue). Original magnification 1000 × (scale bar = 10 µm)

Then, we used double-immunofluorescence staining to verify whether eosinophil infiltration was regulated by Gal-3 in the liver of *S. japonicum*-infected mice. It has been reported that Gal‑3 is predominantly located in the cytoplasm, but it also shuttles into the nucleus and is secreted onto the cell surface and into biological fluids [14]. ECP is a basic and potentially cytotoxic granule protein stored in an inactive form in the granules; it is released from the eosinophil upon activation [15]. Our result showed that a faint intensity of Gal-3 fluorescence (Gal-3^+^, green) and ECP fluorescence (ECP^+^, eosinophils marker, red) was observed in the liver of naive group and lact group. Compared with uninfected controls, a higher intensity of Gal-3 fluorescence and ECP fluorescence was observed in the liver sections of *Sj* group and *Sj* + lact group. Compared with *Sj* group, a lower intensity of Gal-3 fluorescence and a higher intensity of ECP fluorescence were observed in the liver section of *Sj* + lact group (Fig. 4). Thus, blockage of galectin-receptor interaction may induce eosinophil infiltration through downregulation of Gal-3 expression in the liver of *S. japonicum*-infected mice.

### Blockage of galectin-receptor interactions relieved liver fibrosis of *S. japonicum*-infected mice

**Fig. 5:**
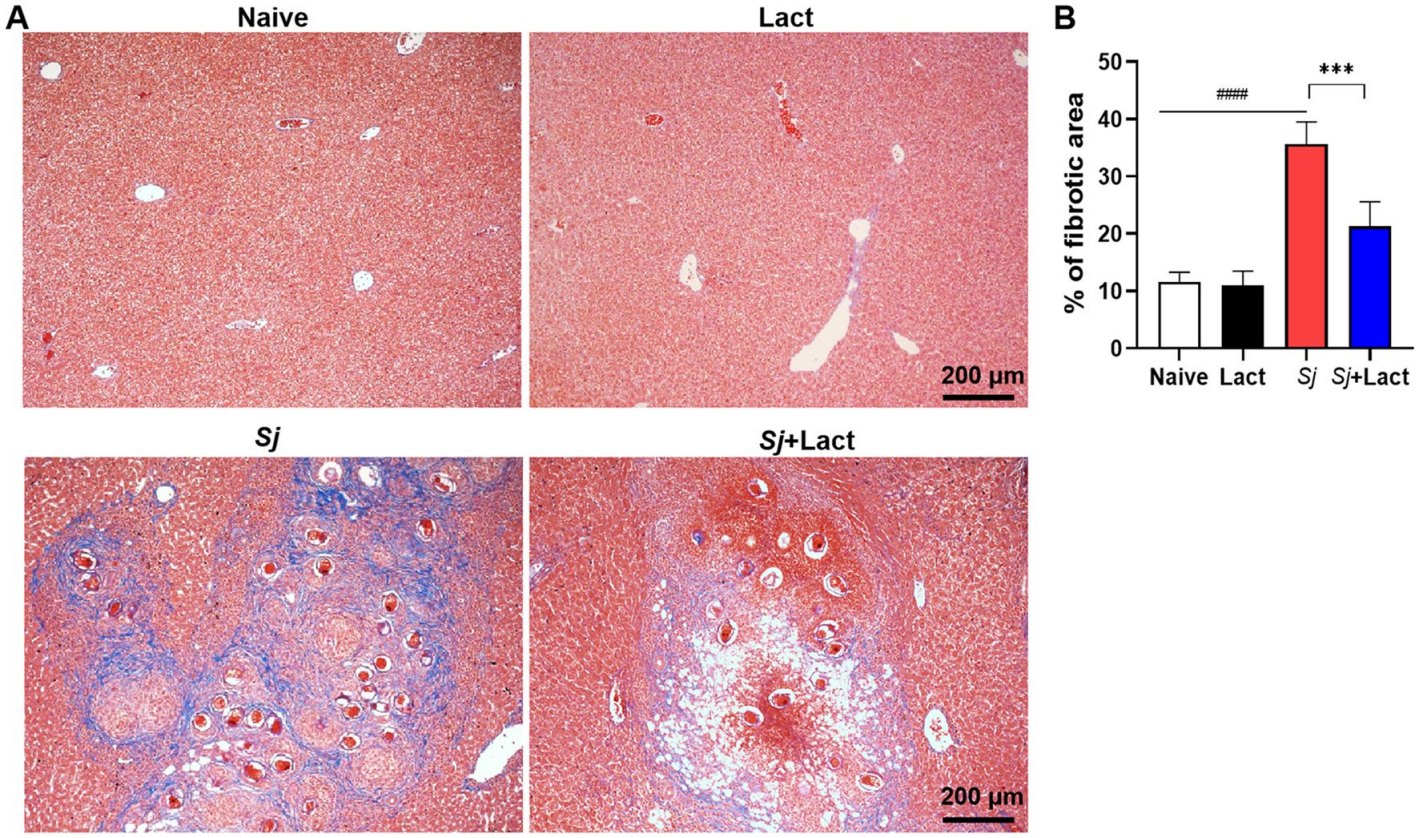
Hepatic fibrosis was relieved in *Schistosoma japonicum*-infected mice with blockage of galectin-receptor. **A** Representative liver granulomas were observed by Massonʼs trichrome staining at 8 weeks p.i. after *S. japonicum* infection. Collagen is in blue color. Original magnification 100 × (scale bar = 200 µm). **B** Collagen deposition positive areas were digitized and analyzed on Image Pro Plus software. Results are expressed as mean ± SD (*n* = 6). ^####^*P* < 0.0001, *Sj* group compared with naive group and lact group; ^***^P < 0.001, *Sj* + lact group compared with *Sj* group

To determine whether blockage of galectin-receptor affects liver fibrogenesis, hepatic collagen deposition was determined by Masson’s trichrome staining. There was a large amount of collagen fiber deposition around the granulomas in the liver section of *Sj* group (Fig. 5A). Compared with *Sj* group, there was significantly decreased fibrotic area in the liver section of *Sj* + lact group (*P* < 0.001, Fig. 5B) (ANOVA; *F* = 83.851; *P* < 0.0001).

**Fig. 6:**
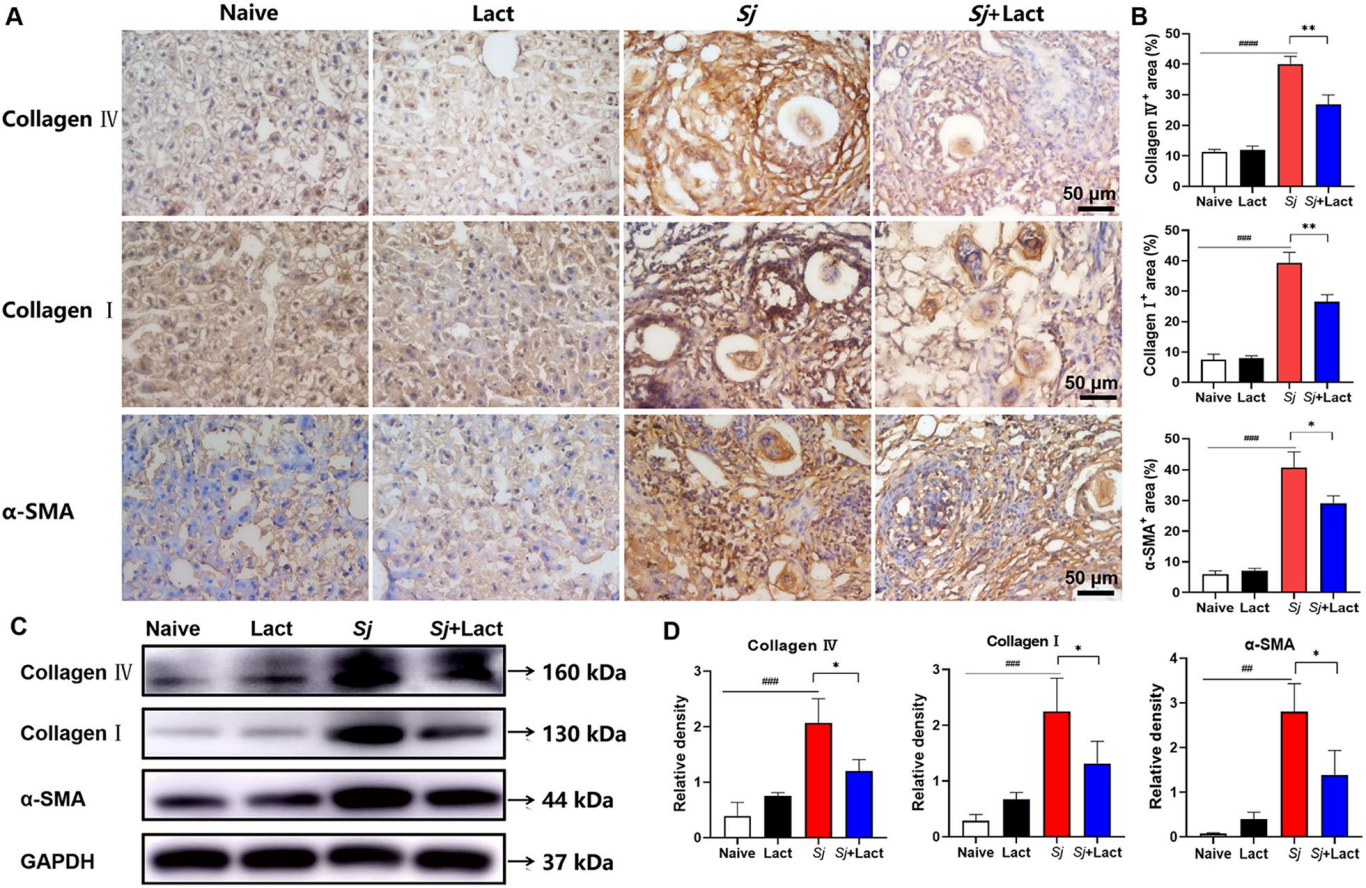
Immunohistochemical staining of α-SMA, collagen I, and collagen IV in the liver. **A** Immunohistochemical staining of α-SMA, collagen I, and collagen IV positive cells in the livers of different groups of mice. Original magnification 400 × (scale bar = 50 µm). **B** α-SMA, collagen I, and collagen IV positive areas were digitized and analyzed by Image Pro Plus software. Data are expressed as mean ± SD (*n* = 4). **C** The protein expression levels of α-SMA, collagen I, and collagen IV in the liver were detected by Western blot. **D** Densitometric analysis of α-SMA, collagen I, and collagen IV normalized to GAPDH and expressed as fold change. Data are expressed as mean ± SD (*n* = 4). ^##^*P* < 0.01, ^###^*P* < 0.001, ^####^*P* < 0.0001, *Sj* group compared with naive group and lact group; ^*^P < 0.05 and ^**^P < 0.01, *Sj* + lact group compared with *Sj* group

The observation by immunohistochemical staining revealed that there were only a few and uniform distribution of α-SMA, collagen I, and collagen IV positive cells (stained brown) observed in the liver sections of naive group and lact group (Fig. 6A). However, compared with uninfected controls, there were increased α-SMA, collagen I, and collagen IV positive areas around egg granulomas in the liver sections of *Sj* group (*P* < 0.0001, *P* < 0.001, and *P* < 0.001, respectively) and *Sj* + lact group (*P* < 0.001, *P* < 0.01, and *P* < 0.001, respectively). Compared with *Sj* group, there were significantly decreased positive areas of α-SMA, collagen I, and collagen IV in the liver section of *Sj* + lact group (*P* < 0.01, *P* < 0.01, and *P* < 0.05, respectively, Fig. 6B) (ANOVA; *F*_α-SMA_ = 193.953, *F*_collagen I_ = 263.689, *F*_collagen IV_ = 257.479; *P* < 0.0001). Consistent results were also found by Western blot, the protein expression levels of α-SMA, collagen I, and collagen IV were significantly decreased in the liver tissue of *Sj* + lact group compared to *Sj* group (*P* < 0.05, Fig. 6C, D) (ANOVA; *F*_α-SMA_ = 50.994, *F*_collagen I_ = 32.637, *F*_collagen IV_ = 41.997; *P* < 0.0001). The results indicate that blockage of galectin-receptor interactions may effectively decrease the expressions of hepatic fibrosis-related proteins in *S. japonicum*-infected mice.

### Blockage of galectin-receptor interactions promoted M1 macrophage polarization in the liver and spleen of *S. japonicum*-infected mice

**Fig. 7:**
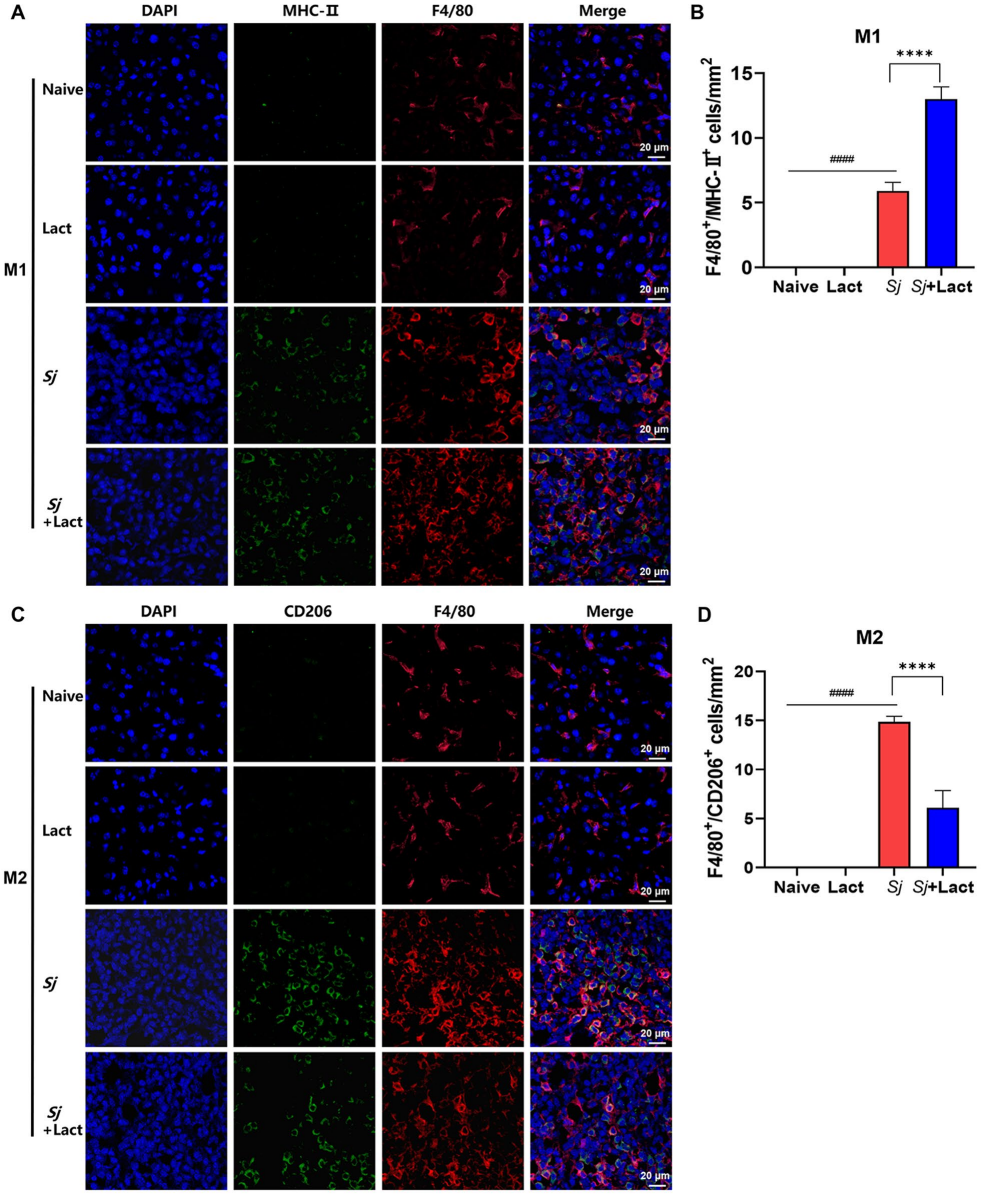
Double immunofluorescence staining of macrophage subtypes in the livers of different groups of mice. **A** Liver sections of different groups of mice were co-stained with anti-F4/80 (macrophage marker, red) and anti-MHC-II (M1 marker, green). DAPI was used to visualize nuclei (blue). Original magnification 600 × (scale bar = 20 µm). **B** F4/80 positive cells and MHC-II positive cells in the liver tissue sections counted in five random fields were quantified. Data are shown as the means ± SD (*n* = 4). **C** Liver sections of different groups of mice were co-stained with anti-F4/80 (red) and anti-CD206 antibodies (M2 marker, green). DAPI was used to visualize nuclei (blue). Original magnification 600 × (scale bar = 20 µm). **D** F4/80 positive cells and CD206 positive cells in the liver tissue sections counted in five random fields were quantified. Data are shown as the means ± SD (*n* = 4). ^####^*P* < 0.0001, *Sj* group compared with naive group and lact group; ^****^P < 0.001, *Sj* + lact group compared with *Sj* group

The expressions of MHC-II (a phenotypic marker for M1 macrophages) and CD206 (a phenotypic marker for M2 macrophages) in the liver of different groups of mice were compared using immunofluorescence staining, and macrophages were detected by staining with anti-F4/80 antibody. A few F4/80 positive cells (F4/80^+^ cells, red fluorescence) were observed in the liver sections of naive group and lact group, but many F4/80^+^ cells were observed in the liver sections of *Sj* group and *Sj* + lact group (Fig. 7A, C). Meanwhile, compared with naive group and lact group, more MHC-II positive cells (MHC-II^+^ cells, green fluorescence) or CD206 positive cells (CD206^+^ cells, green fluorescence) and merged with F4/80^+^ cells in yellow color were observed in the liver sections of *Sj* group and *Sj* + lact group (Fig. 7A, C). The numbers of MHC-II^+^ cells and CD206^+^ cells in the liver tissues of *Sj* group and *Sj* + lact group were significantly higher than those of naive group and lact group (ANOVA; *F*_MHC-II_ = 462.657, *F*_CD206_ = 229.691; *P* < 0.0001, Fig. 7B, D). Compared with *Sj* group, the number of MHC-II^+^ cells was significantly increased (*P* < 0.0001), whereas the number of CD206^+^ cells was significantly decreased (*P* < 0.0001) in the liver tissue of *Sj* + lact group.

**Fig. 8:**
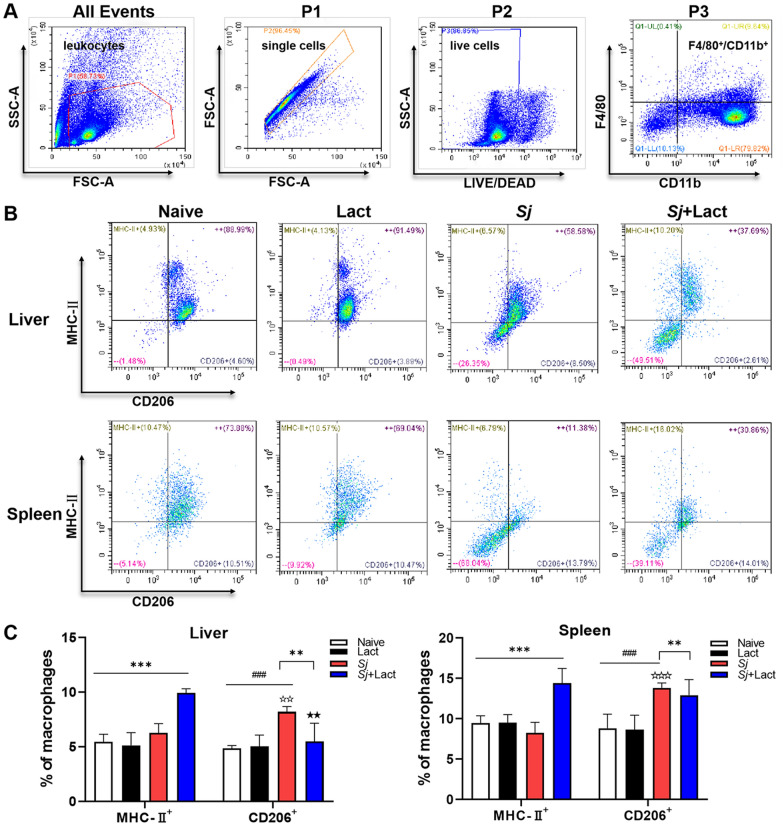
Flow cytometry for M1/M2 macrophages in the liver and spleen of different groups of mice. **A** Gating strategy of the flow cytometry data. P1 represented the leukocyte subsets gated from all the events; P2 represented the single cells gated from the P1; P3 represented the live cells gated from the P2; F4/80^+^/CD11b^+^ cells (Q1-UR, top right corner) were the macrophage subtypes gated from the cells of the P3. **B** Representative data of the M1/M2 macrophage subtypes in different groups. The quadrants show MCH-II^+^/CD206^–^ cells (MCH-II^+^, upper left), MCH-UII^+^/CD206^+^ cells (^+ +^, upper right), MCH-II^–^/CD206^–^ cells (^– –^, lower left), and MCH-II^–^/CD206^+^ cells (CD206^+^, lower right). **C** The percentage of MCHII^+^/CD206^–^ cells and MCH-II^–^/CD206^+^ cells in the liver and spleen. Data are shown as the means ± SD (*n* = 4). ^###^*P* < 0.001, *Sj* group compared with naive group and lact group; ^**^P < 0.01 and ^***^P < 0.001, *Sj* + lact group compared with naive group, lact group, and *Sj* group. ^☆☆^*P* < 0.01 and ^☆☆☆^*P* < 0.001, MCH-II^+^ cells compared with CD206^+^ cells in *Sj* group; ^★★^*P* < 0.01, MCH-II^+^ cells compared with CD206^+^ cells in *Sj* + lact group

The gating strategy of flow cytometry data used in the liver samples of *Sj* + lact group was as below; this was also used in all liver and spleen samples of different groups: (i) gated the leukocyte subsets from all cells according to the parameters of SSC-A/FSC-A, namely P1; (ii) gated the single cells from P1 according to FSC-A/FSC-A parameters, namely P2; (iii) gated the live cells from P2 according to SSC-A/LIVE/DEAD parameters, namely P3; (iv) F4/80^+^/CD11b^+^ cells (Q1-UR, top right corner) were gated from the cells of the P3, which were macrophage subtypes (Fig. 8A). Based on the above gating data, the M1/M2 macrophage subtypes of antibody-labeled MHC-II and CD206 cells in the livers and spleens of mice of different groups were further analyzed (Fig. 8B). Compared with naive group and lact group, there were significantly increased CD206 cells in the liver and spleen of *Sj* group (*P* < 0.001); however, the proportion of MHC-II cells had no significant change. Compared with *Sj* group, there were significantly increased MHC-II cells in the liver (*P* < 0.001) and spleen (*P* < 0.001) and significantly decreased CD206 cells in the liver (*P* < 0.01) of *Sj* + lact group (ANOVA; *F*_liver MHC-II_ = 42.127, *F*_liver CD206_ = 13.868, *F*_spleen MHC-II_ = 25.253, *F*_spleen CD206_ = 16.672; *P* < 0.0001, Fig. 8C). These findings suggest that *S. japonicum* infection induced increased M2 macrophages, while blockage of galectin-receptor interactions resulted in increased M1 macrophages and decreased M2 macrophages in the liver of *Sj* + lact mice.

**Fig. 9:**
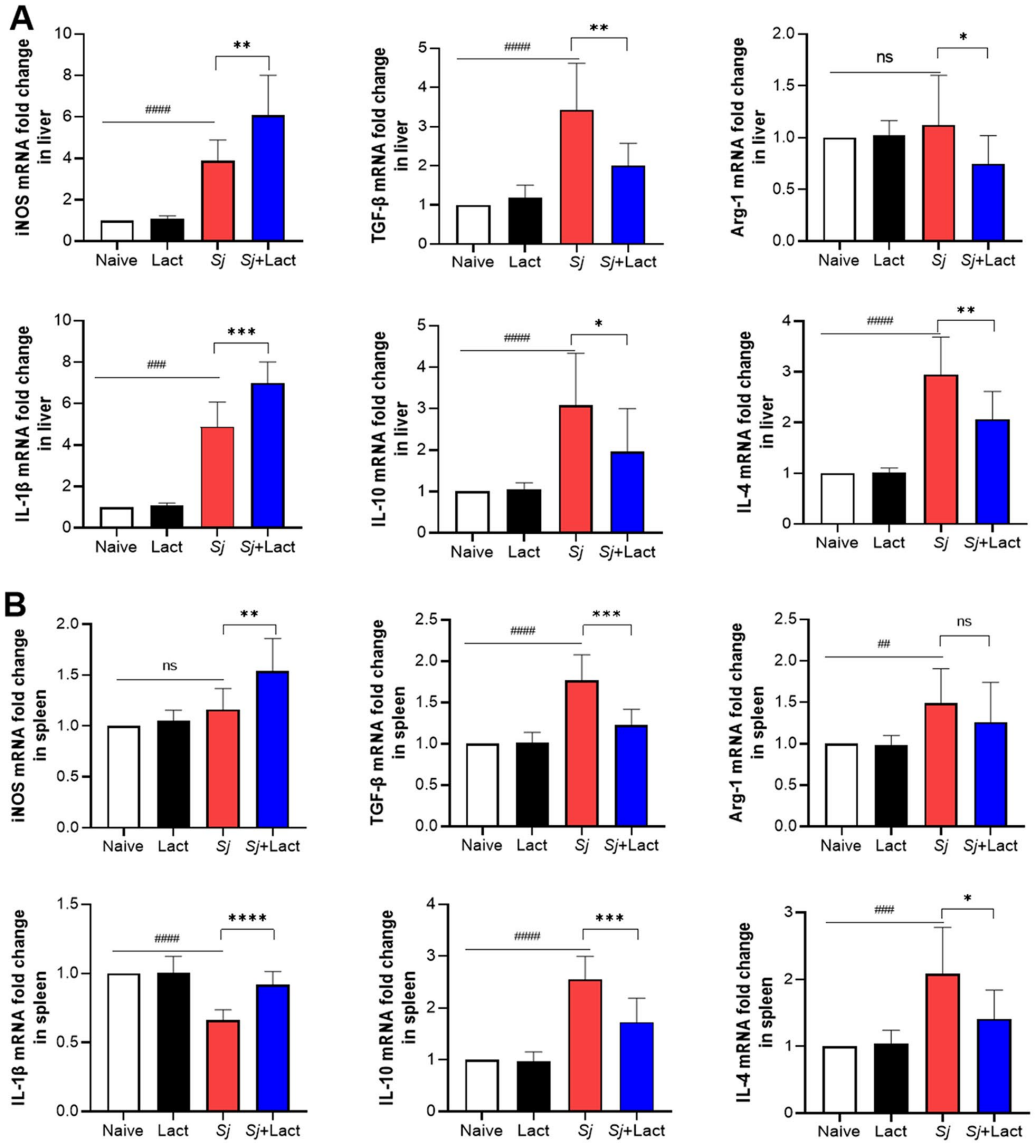
mRNA expression levels of M1 and M2 macrophage cytokines of different groups of mice. **A** mRNA expression levels of M1 and M2 macrophage cytokines in the liver. **B** mRNA expression levels of M1 and M2 macrophage cytokines in the spleen. Values are means from triplicate measurements, and data are presented as means ± SD (*n* = 10); Y axis represents the mRNA expression levels of different genes. ns: no significance; ^##^*P* < 0.01, ^###^*P* < 0.001, and ^####^*P* < 0.0001, *Sj* group compared with naive group and lact group; ^*^P < 0.05, ^**^P < 0.01, ^***^P < 0.001, and ^****^P < 0.0001, *Sj* + lact group compared with *Sj* group

We further evaluated whether α-lactose treatment induced changes in M1 and M2 macrophage cytokines in the liver and spleen of mice with *S. japonicum* infection. Compared with naive group and lact group, there were significantly increased mRNA expression levels of iNOS (*P* < 0.0001), IL-1β (*P* < 0.001), TGF-β (*P* < 0.0001), IL-10 (*P* < 0.0001 and *P* < 0.01, respectively), and IL-4 (*P* < 0.0001) in the livers of *Sj* group and *Sj* + lact group (ANOVA; *F*_iNOS_ = 50.657, *F*_IL-1β_ = 141.254, *F*_TGF-β_ = 26.876, *F*_IL-10_ = 14.213, *F*_IL-4_ = 40.989; *P* < 0.0001, Fig. 9A); significantly increased mRNA levels of TGF-β (*P* < 0.0001), IL-10 (*P* < 0.0001), and IL-4 (*P* < 0.001) in the spleen of *Sj* group, and significantly increased iNOS mRNA level (*P* < 0.01) in the spleen of *Sj* + lact group (ANOVA; *F*_iNOS_ = 15.191, *F*_IL-1β_ = 42.234, *F*_TGF-β_ = 34.811, *F*_IL-10_ = 49.043, *F*_IL-4_ = 14.040; *P* < 0.0001, Fig. 9B). However, compared with *Sj* group, there were significantly increased mRNA levels of iNOS (*P* < 0.001) and IL-1β (*P* < 0.001 and *P* < 0.0001, respectively) in the liver and spleen, significantly decreased mRNA levels of TGF-β (*P* < 0.01), IL-10 (*P* < 0.05), Arg-1 (*P* < 0.05), and IL-4 (*P* < 0.01) in the liver, and significantly decreased mRNA levels of TGF-β (*P* < 0.001), IL-10 (*P* < 0.001), and IL-4 (*P* < 0.05) in the spleen of *Sj* + lact group (Fig. 9). The results indicated that blockage of galectin-receptor interactions may increase M1 macrophage response and decrease M2 macrophage response in the liver and spleen of *S. japonicum*-infected mice.

### Correlation analysis between mRNA expressions of Gal-3 and M1/M2 macrophage cytokines in the liver of *S. japonicum*-infected mice

**Fig. 10:**
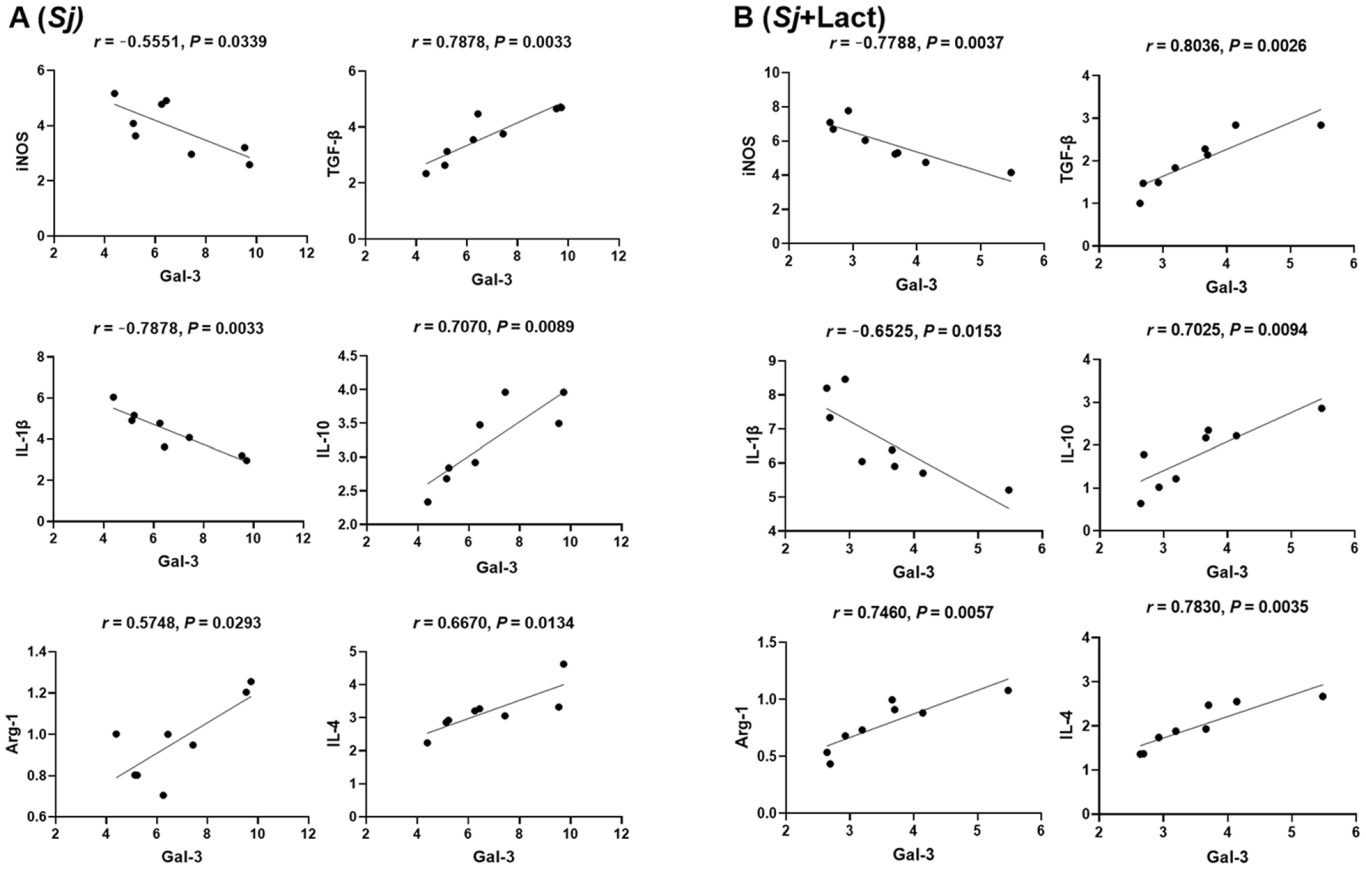
Correlation analysis between the mRNA expression levels detected in the livers of different groups of mice. Correlation analysis between the mRNA expression levels of Gal-3 and iNOS/IL-1β/TGF-β/IL-10/Arg-1/IL-4 in the livers of *Sj* group **A** and *Sj* + lact group **B** (n = 8). The *r* value generates the theoretical line of best fit, and the *P* value indicates the significance of the correlation

Only significant correlations between the mRNA levels of Gal-3 and M1/M2 macrophage cytokines in the livers of *Sj* group and *Sj* + lact group were provided here. There were significant negative correlations between the mRNA levels of Gal-3 and iNOS (Pearson' s correlation coefficient, *r* = 0.5551, *P* = 0.0339) and Gal-3 and IL-1β (*r* = 0.8464, *P* = 0.0012) and significant positive correlations between the mRNA levels of Gal-3 and TGF-β (*r* = 0.7878, *P* = 0.0033), Gal-3 and IL-10 (*r* = 0.7070, *P* = 0.0089), Gal-3 and Arg-1 (*r* = 0.5748, *P* = 0.0293), and Gal-3 and IL-4 (*r* = 6670, *P* = 0.0134) in the liver of *Sj* group (Fig. 10A), while there were significant negative correlations between the mRNA levels of Gal-3 and iNOS (*r* = 0.7788, *P* = 0.0037), and Gal-3 and IL-1β (*r* = 0.6525, *P* = 0.0153) and significant positive correlations between the mRNA levels of Gal-3 and TGF-β (*r* = 0.8036, *P* = 0.0026), Gal-3 and IL-10 (*r* = 0.7025, *P* = 0.0094), Gal-3 and Arg-1 (*r* = 0.7460, *P* = 0.0057), and Gal-3 and IL-4 (*r* = 0.7830, *P* = 0.0035) in the liver of *Sj* + lact group (Fig. 10B).

### Blockage of galectin-receptor interaction promoted macrophage autophagy through the Akt/mTOR signaling pathway in the liver of* S. japonicum*-infected mice

**Fig. 11:**
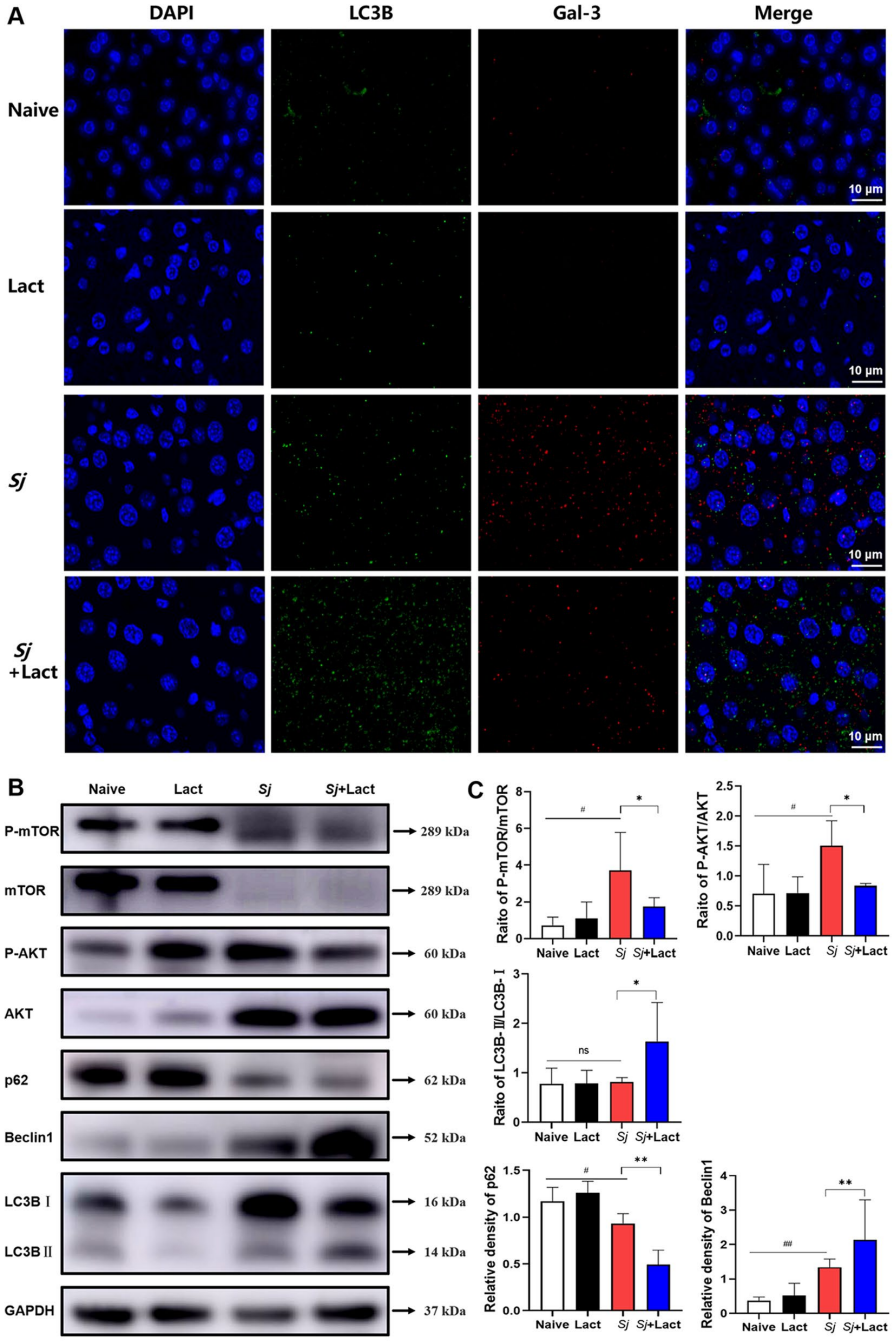
Effects of α-lactose on autophagy Akt/mTOR signaling pathway in the livers of different groups of mice. **A** Liver sections of different groups of mice were co-stained with anti-Gal-3 (red) and anti-LC3B (autophagy marker, green). DAPI was used to visualize nuclei (blue). Original magnification 1000 × (scale bar = 10 µm). **B** Western blot analysis for p-mTOR, mTOR, p-AKT, AKT, p62, Beclin1, LC3B, and GAPDH. **C** p-mTOR, p-AKT, and LC3B-II band density normalized to mTOR, AKT, and LC3B-I, respectively; the relative densitometry of p62 and Beclin1 (relative to the endogenous control, GAPDH) expressed as fold change. The values are expressed as mean ± SD (*n* = 4). ns: no significance; ^#^*P* < 0.05 and ^##^*P* < 0.01, *Sj* group compared with naive group and lact group; ^*^P < 0.05 and ^**^P < 0.01, *Sj* + lact group compared with *Sj* group

Study has reported that Gal-3, as a negative regulator of autophagy, can decrease autophagy activity [16]. To verify whether autophagy is regulated by Gal-3 in the liver of *S. japonicum*-infected mice, immunofluorescence staining showed that faint intensities of LC3B fluorescence and Gal-3 fluorescence were observed in the livers of naive group and lact group. Compared with uninfected mice, there were higher intensities of LC3B fluorescence and Gal-3 fluorescence in the liver of *Sj* group. Compared with *Sj* group, the intensity of LC3B fluorescence was stronger and the intensity of Gal-3 fluorescence was lower in the liver of *Sj* + lact group (Fig. 11A). Thus, blockage of galectin-receptor interaction may promote autophagy through downregulating Gal-3 expression in the liver of *S. japonicum*-infected mice.

Western blot was performed to explore *S. japonicum* infection-induced autophagy signaling pathway. The results showed that, compared with *Sj* group, there were significantly decreased p62 protein expression (*P* < 0.01), significantly decreased protein expression ratio of phospho-mTOR/mTOR (*P* < 0.05), and significantly decreased protein expression ratio of phospho-AKT/AKT (*P* < 0.05) while significantly increased Beclin1 protein expression (*P* < 0.01) and significantly increased protein expression ratio of LC3B-II/LC3B-I (*P* < 0.05) in the liver of *Sj* + lact group at 8 weeks p.i. (ANOVA; *F*_p62_ = 29.132, *P* < 0.0001; *F*_phospho-mTOR/mTOR_ = 5.112, *P* < 0.05; *F*_phospho-AKT/AKT_ = 7.234, *P* < 0.01; *F*_Beclin1_ = 6.898, *P* < 0.01; *F*_LC3B-II/LC3B-I_ = 3.551, *P* < 0.05, Fig. 11B, C).

**Fig. 12:**
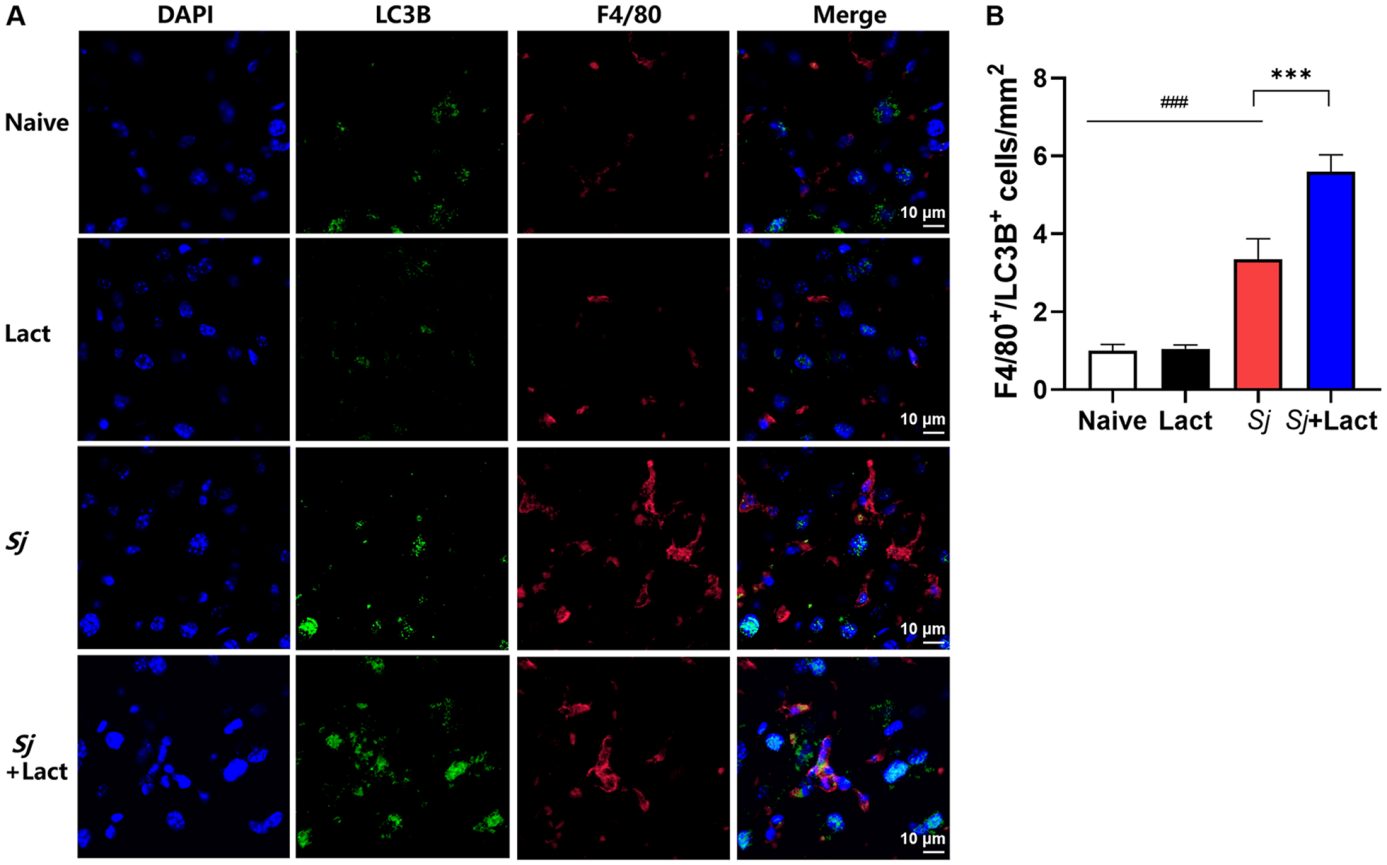
Double immunofluorescence staining of F4/80 and LC3B in the liver tissues of different groups of mice. **A** Liver sections of different groups of mice were co-stained with anti-F4/80 (macrophage marker, red) and anti-LC3B (autophagy marker, green). DAPI was used to visualize nuclei (blue). Original magnification 1000 × (scale bar = 10 µm). **B** F4/80^+^/LC3B^+^ cells in the liver tissue sections counted in five random fields were quantified. Data are shown as the means ± SD (*n* = 4). ^###^*P* < 0.001, *Sj* group compared with naive group and lact group; ^***^P < 0.001, *Sj* + lact group compared with *Sj* group

According to the above results, we found that blockage of galectin-receptor interaction could promote autophagy in the liver of *Sj* + lact group; however, which kind of cells undergoes autophagy needs to be investigated. By immunofluorescence staining, a few F4/80 positive cells (F4/80^+^ cells with red fluorescence) were observed in the liver sections of naive group and lact group, but many F4/80^+^ cells were observed in the liver sections of *Sj* group and *Sj* + lact group (Fig. 12A). Compared with naive group and lact group, more double-labeled cells (F4/80^+^/LC3B^+^ cells) merged in yellow color were counted in the liver section of *Sj* group (*P* < 0.001). Compared with *Sj* group, the number of F4/80^+^/LC3B^+^ cells was significantly increased (*P* < 0.001) in the liver section of *Sj* + lact group (ANOVA; *F* = 153.973; *P* < 0.001, Fig. 12B). The results suggest that macrophage autophagy may be involved in regulating liver pathogenesis of *S. japonicum*-infected mice.

## Discussion

Schistosomiasis is a parasitic disease caused by trematode blood flukes of the genus *Schistosoma*. It affects almost 240 million people worldwide, and > 700 million people live in endemic areas [2]. The formation of granulomata around eggs of *S. japonicum* deposited in the liver is related to hepatic fibrogenesis in schistosomiasis japonica [17]. However, the mechanism of hepatic fibrosis caused by *S. japonicum* egg granuloma remains not well defined.

Galectins are lectins with a highly conserved carbohydrate recognition domain (CRD) and affinity for β galactose-containing oligosaccharides [18]. Gal-3 is predominantly located in the cytoplasm with multiple functions including regulation of inflammation, cell growth, signaling, chemotaxis, and cell–matrix interactions, etc. [19]. Gal-3 has been described as a biomarker associated with fibrosis and inflammation in patients with heart failure [20]. In the present study, serum ALT and AST levels, the liver and spleen indexes, liver and spleen histopathological score, the grade of hepatic fibrosis, and Gal-3 expression level in the liver were significantly decreased in *Sj* + lact group compared with *Sj* group. It has been reported that Gal-3 is involved in Chagas disease and mediating cardiac tissue damage as well as in immune responses against *Trypanosoma cruzi* experimental infection; lack of Gal-3 prevents cardiac fibrosis [21]. Genetic deletion of Gal-3 prevented cardiac damage, adverse remodeling, and dysfunction, associated with reduced cardiac oxidative stress and fibrosis in C57 Gal-3 knockout mice treated with doxorubicin [22]. The role of Gal-3 in fibrosis and immune response has been extensively studied. In the present study, our results found that Gal-3 was strongly involved in hepatic immunopathology induced by *S. japonicum* infection, and blockage of galectin-receptor interactions resulted in relieved liver function and decreased liver pathology and fibrosis in *S. japonicum*-infected C57BL/6 mice, indicating the potential therapeutic effect of Gal-3 in fibrotic pathogenesis caused by *S. japonicum* infection.

It has been reported that the eggs of *S. japonicum* and *S. mansoni* induced local granulomas inflammatory response, and granulomas mainly consist of lymphocytes, macrophages, and eosinophils [17]. The role of eosinophils in the pathogenesis of schistosomiasis remains controversial [23]. Study has demonstrated that eosinophils play a role in limiting the pathology associated with human schistosomiasis mansoni [24], and ECP may be an important protein both in the immune response against *S. mansoni* and in the development of periportal fibrosis [24]. However, experiments using *S. mansoni*-infected mouse models of eosinophil lineage ablation demonstrated that eosinophils had no impact on granuloma number, size, or fibrosis, and eosinophil ablation also had no effect on worm burden or egg deposition [25]. Therefore, eosinophils may make unknown immunomodulatory contributions to liver fibrosis of mice infected with *S. mansoni* [25]. In the absence of Gal-3, *S. mansoni* egg granulomas in chronic-phase are smaller in diameter, with thinner collagen fibers [26]. *Schistosoma mansoni*-infected Gal-3^–/–^ mice have fewer macrophages and B lymphocytes but increased eosinophil absolute number in the periphery compared with the infected wild-type mice [26]. The inflammatory cell composition around *S. mansoni* egg granulomas in Lgals3^–/–^ mice presents dominance of eosinophils in liver granulomas, and there is significant inflammatory infiltration as well as phagocytes (specifically Kupffer cells) and numerically reduced and diffuse matrix extracellular deposition in fibrotic areas in the liver of Lgals3^−/−^ infected mice compared to Lgals3^+/+^ mice [7]. In the present study, there were decreased Gal-3 expression, increased eosinophil infiltration and ECP expression, and increased mRNA levels of ECP, EPO, IL-5, CCL11, and CCR3 in the liver of *Sj* + lact group compared to *Sj* group. However, hepatic histopathological changes and fibrosis were significantly alleviated, accompanied by significantly decreased α-SMA, collagen I, and collagen IV in the liver of *Sj* + lact group. Our data further demonstrated that blockage of galectin-receptor interactions increases eosinophil numbers and eosinophil degranulation in the liver of *S. japonicum*-infected mice, which has a great impact on the immunomodulation of granulomatous inflammation and decreases hepatic lesions induced by egg deposition of *S. japonicum*.

Gal-3 is an immune regulator and induces the immune response against *S. mansoni* infection, and the absence of Gal-3 interfered with the function of the resident liver macrophages and Kupffer cells [7]. Because Gal-3 is highly expressed in inflammatory cells surrounding liver granulomas, it is considered to favor the interactive network between macrophages and immunogenic carbohydrates [27]. Gal-3 is highly expressed by Kupffer cells in liver around *S. mansoni* eggs [7]. Compared with *S. mansoni*-infected Lgals3^+/+^ mice, there was significant inflammatory infiltration with lower levels of α-SMA and eotaxin and higher levels of IL-4 in *S*. *mansoni*-infected Lgals3^–/–^ mice at 90 days p.i. The reduction of macrophages and decreased phagocytic activity of local Kupffer cells in *S*. *mansoni*-infected Lgals3^–/–^ mice suggest that Gal-3 is necessary for fibrotic concentric granulomas and myofibroblast activation in *S. mansoni*-infected mice [7]. In the present study, blockage of galectin-receptor interactions resulted in decreased M2 macrophages and increased M1 macrophages in the liver of *Sj* + lact mice. It has been reported that iNOS and IL-1β are M1 macrophage-associated genes, and TGF-β, Arg1, IL-4, and IL-10 are M2 macrophage-associated genes [28]. We evaluated M1 and M2 macrophage cytokines in the liver and spleen of mice of different groups; there were significantly increased levels of iNOS and IL-1β in the liver and spleen, and significantly decreased levels of TGF-β, IL-10, Arg-1, and IL-4 in the liver and significantly decreased levels of TGF-β, IL-10,  and IL-4 in the spleen of *Sj* + lact group compared to *Sj* group. Additionally, our results showed that there were significant negative correlations between the mRNA levels of Gal-3 and M1 macrophage cytokines (iNOS/IL-1β) and significant positive correlations between the mRNA levels of Gal-3 and M2 macrophage cytokines (TGF-β/Arg-1/IL-10/IL-4) in the liver of *Sj* + lact group. Our data demonstrated that blockage of galectin-receptor interactions increases M1 macrophage response and decreases M2 macrophage response in the liver and spleen of *S. japonicum*-infected mice, indicating that Gal-3 is associated with macrophage polarization.

Macrophage autophagy has an important regulatory role in downregulation of hepatic immunopathology in different diseases, and macrophages play a critical role in *S. japonicum* egg-triggered immunopathology in host liver [9]. Alveolar macrophages are significant sources of Gal-3, which drives inflammation and the development of pulmonary fibrosis and reduction in Gal-3 expression in alveolar macrophages and neutrophils decreasing inflammation and neutrophil recruitment into the interstitium of lung [29]. In the present study, by immunofluorescence staining, more F4/80^+^/LC3B^+^ cells were observed in the liver of *Sj* + lact group compared to *Sj* group. It has been reported that Gal-3, as a negative regulator of autophagy, can decrease autophagy activity [16]. Our result is consistent with this study.

A study has reported that the PI3K/Akt/mTOR signal has a critical role in the biology of *S. mansoni*; it is involved in glucose uptake by *S. mansoni*, the reproduction of *S. mansoni*, and worm survival [30]. However, whether the Akt/mTOR pathway can impact on liver immunopathology caused by *S. japonicum* infection through regulating macrophage autophagy has not been reported. *Enterococcus faecalis* lipoteichoic acid may increase macrophage autophagy by inhibiting the PI3K/Akt/mTOR pathway [31]. Doxazosin inhibits autophagy by activation of the PI3K/Akt/mTOR signaling pathway in hepatic stellate cells [32]. In the present study, our results showed that Gal-3 expression was decreased while LC3B expression was increased in the liver of *Sj* + lact group compared to *Sj* group. There were decreased p62 protein expression and decreased protein expression ratios of phospho-mTOR/mTOR and phospho-AKT/AKT, while increased Beclin1 protein expression and increased protein expression ratio of LC3B-II/LC3B-I in the liver of *Sj* + lact group at 8 weeks p.i. compared with *Sj* group, indicating that blockage of galectin-receptor interaction (e.g. through downregulating Gal-3 expression) may reduce *S. japonicum*-induced liver fibrosis by promoting autophagy by inhibiting AKT/mTOR signaling pathway activation. Gal-3 knockdown may enhance autophagy by inhibiting the Gal-3/Akt/mTOR pathway, which helps ameliorate renal fibrosis [33]. We proposed that autophagy signaling pathway could be involved in the pathogenesis of schistosomiasis japonica. Further study focused on macrophage autophagy could elucidate the pathological mechanisms of liver fibrosis caused by *S. japonicum* infection.

In conclusion, we used mouse models to investigate whether galectins were involved in the immunopathological mechanisms of liver fibrosis caused by *S. japonicum* infection by blocking galectin-receptor interactions with α-lactose; our data suggested that Gal-3 plays a pivotal role during *S. japonicum* infection, which leads to attenuated liver functional damage and liver fibrosis by promoting eosinophil infiltration, M1 macrophage polarization, and macrophage autophagy. Thus, use of Gal-3 inhibition may be a therapeutic approach to prevent the early liver fibrosis caused by *S. japonicum* infection.

### Supplementary Information

Supplementary material 1.

## Data Availability

The data supporting the conclusions of this article have been included in the article/supplementary material.

## Abbreviations

ALT: Alanine aminotransferase
AST: Aspartic aminotransferase
BSA: Bovine serum albumin
CCL11: C–C motif chemokine 11
CCR3: CC chemokine receptor 3
CRD: Carbohydrate recognition domain
DAPI: 4,6-Diamidino-2-phenylindole
ECP: Eosinophil cationic protein
EPO: Eosinophil peroxidase
Gal: Galectin
H&E: Hematoxylin-eosin
HSCs: Hepatic stellate cells
i.P.: Intraperitoneal
qRT-PCR: Quantitative real-time reverse transcription-polymerase chain reaction
Sj: *S. japonicum*
α-SMA: Alpha-smooth muscle actin
